# Tumor progression is effectively suppressed by miR-195 through direct targeting of YAP1

**DOI:** 10.3389/fonc.2026.1858597

**Published:** 2026-07-23

**Authors:** Xiao-Wei Wang, Jin-Chao Song, Ming-Ming Tang, Yi-Yu He, Zhi-Gang Cai

**Affiliations:** 1 Department of Cardio-Thoracic Surgery, Changhai Hospital, Shanghai, China; 2 Department of Anesthesiology, Shidong Hospital of Shanghai, University of Shanghai for Science and Technology, Shanghai, China; 3 Department of Cardio-Thoracic Surgery, Naval Medical Center of PLA, Shanghai, China

**Keywords:** hippo pathway, miR-195, miRNA, tumor suppression, YAP1

## Abstract

**Background:**

Yes-associated protein 1 (YAP1) dysregulation is frequently observed in various human cancers. This study aimed to identify microRNAs (miRNAs) capable of suppressing YAP1 expression and to elucidate their functional roles in tumor progression.

**Methods:**

We screened five candidate miRNAs using a luciferase reporter assay containing the YAP1 3’ untranslated region (3’ UTR). Functional validation was performed using gain- and loss-of-function experiments in HCT116 and A549 cell lines. The *in vivo* effects of miR-195 were evaluated using a xenograft model.

**Results:**

Among the tested miRNAs, miR-195 demonstrated the most potent suppression of YAP1 3’ UTR activity. This interaction was direct and specific, as a mutation in the YAP1 3’ UTR seed sequence abolished miR-195-mediated repression. Consequently, miR-195 overexpression significantly reduced endogenous YAP1 protein levels and suppressed the expression of its downstream target, CTGF. Functionally, miR-195 overexpression inhibited cell viability and reduced colony formation by approximately 40% in HCT116 cells, while miR-195 knockdown promoted proliferation. Rescue experiments confirmed that the anti-proliferative effects of miR-195 were dependent on YAP1 suppression. *In vivo*, stable overexpression of miR-195 significantly attenuated tumor growth in xenograft models. Furthermore, analysis of clinical data revealed that downregulation of miR-195 is prevalent in multiple tumor types and correlates with poor patient survival.

**Conclusion:**

Our findings establish miR-195 as a critical tumor suppressor that directly targets YAP1, highlighting its potential as a therapeutic target in cancer treatment.

## 1 Introduction

The Hippo-YAP signaling pathway is an evolutionarily conserved kinase cascade that governs organ size, cell fate determination, and tissue homeostasis (1). This pathway comprises a core kinase module, including the mammalian STE20-like kinases (MST1/2) and the large tumor suppressors (LATS1/2), which form complexes with adaptor proteins Salvador homologue 1 (SAV1) and MOB kinase activator 1 (MOB1A/B) (2). Activation of the Hippo cascade triggers LATS1/2-mediated phosphorylation of YAP1 and TAZ (3). Consequently, phosphorylated YAP1/TAZ are sequestered in the cytoplasm and subjected to ubiquitin-mediated degradation, thereby precluding their nuclear translocation. Conversely, inactivation of the Hippo pathway permits unphosphorylated YAP1/TAZ to translocate into the nucleus, where they interact with transcription factors such as the TEAD family to drive the expression of genes that promote cell proliferation and survival (4, 5).

Dysregulation of the Hippo pathway, leading to YAP1/TAZ accumulation, is a frequent event in human cancers, including colorectal, liver, and lung carcinomas (6–8). YAP1 overexpression drives uncontrolled cellular proliferation and accelerates cell cycle progression (9, 10). Conversely, YAP1 depletion suppresses tumorigenic characteristics *in vitro* and *in vivo* (11, 12). As YAP1 lacks a DNA-binding domain, it functions as a transcriptional co-activator by forming complexes with partners such as the TEAD family of transcription factors (13–15). This YAP1-TEAD complex regulates the expression of pro-proliferative genes (e.g., *CTGF* and *CYR61*) and interacts with other nuclear proteins, including SMADs and p73, to facilitate tumor progression (14, 16–18).

MicroRNAs (miRNAs) are endogenous, non-coding RNAs that regulate gene expression post-transcriptionally by binding to the 3’ UTRs of target mRNAs, leading to mRNA degradation or translational repression (19, 20). It is estimated that miRNAs regulate approximately 60% of the human genome and are implicated in nearly all biological processes, including tumorigenesis (21–24). Dysregulation of miRNAs is a hallmark of cancer, where they can function as either oncogenes or tumor suppressors (25).

Recent studies indicate that miRNAs play crucial roles in modulating the Hippo-YAP pathway. For instance, miR-135b promotes breast cancer growth by targeting LATS2 (26), while miR-9-3p and miR-125a suppress tumor progression by targeting TAZ (27, 28). Furthermore, several miRNAs, including miR-27, miR-15/16, and miR-199a-3p, have been shown to target YAP1 directly to inhibit tumor growth and metastasis (29–31). Previous studies have also reported that miR-199 can regulate YAP1 expression, though its functional role in tumorigenesis may be context-dependent (29–31).

In this study, we systematically evaluated the suppressive effects of multiple miRNAs on YAP1 expression. Among them, miR-195 exhibited the most robust inhibitory effect on YAP1 expression. We further demonstrated that miR-195 inhibits tumor cell proliferation and *in vivo* tumor growth by directly targeting YAP1, suggesting its potential as a therapeutic target.

## 2 Materials and methods

### 2.1 Plasmid construction

MiRNA precursors (pre-miR-16, pre-miR-27a, pre-miR-195, and pre-miR-199) were amplified via PCR from genomic DNA using specific primers. HindIII pre-miR-16 F: TCTAAGCTTTTGCTGCTCTAGAAATTTAAG, XhoI pre-miR-16 R: CTTCTCGAGTAGGCCATTGTGTACAAATTA, size: 350 bp; HindIII pre-miR-27a F: AGGAAGCTTGAGGCCAGGCAGCAGGAT, XhoI pre-miR-27a R: AGGCTCGAGGATTTCCAACCGACCCTGAG, size: 236 bp; HindIII pre-miR-195 F: CACAGATCTGGGACAGGAGACACTGGAAAG, XhoI pre-miR-195 R: CCCCTCGAGCTGAGCCTTCCACCTCTGACCC, size: 475 bp; HindIII pre-miR-199 F: TCTAAGCTTAAGAGTGGTGGTTTCCTTGG, XhoI pre-miR-199 R: AGGCTCGAGGGGTGGTGGAAAATGACACT, Size: 248 bp. The PCR products were digested with HindIII and XhoI and ligated into the pAdtrack plasmid. For the YAP1 3’ UTR reporter, the YAP1 3’ UTR sequence was amplified and cloned into the psiCheck2 plasmid downstream of the Renilla luciferase gene. Site-directed mutagenesis was performed to disrupt the miR-195 seed sequence binding site.

### 2.2 Cell culture and transfection

293T, A549, and HCT116 cells were cultured in DMEM or McCoy’s 5A medium supplemented with 10% FBS. Cells were transfected using Lipofectamine 2000 (Invitrogen) according to the manufacturer’s instructions. For lentiviral infections, cells were infected at an MOI of 50 and selected with Puromycin (10 μg/ml) to establish stable cell lines.

### 2.3 RNA extraction and qRT-PCR

Total RNA was extracted using TRIzol reagent (Invitrogen). cDNA was synthesized using the M-MLV reverse transcriptase. Specific stem-loop primers were used for miRNA detection, and random primers were used for mRNA. Real-time PCR was performed using SYBR Green (TOYOBO). U6 snRNA was used as an endogenous control for miRNAs. The relative expression levels were calculated using the 2^−ΔΔCt^ method. Primer sequences for miRNA RT-PCR were as follows: MiR-16 RT primer: GTCGTATCCAGTGCAGGGTCCGAGGTATTCGCACTGGATACGACCGCCAA, miR-16 F: CGCGCTAGCAGCACGTAAAT, and miR-16 R: GTGCAGGGTCCGAGGT; miR-27a RT primer: GTCGTATCCAGTGCAGGGTCCGAGGTATTCGCACTGGATACGACGCGGAA, miR-27a F: CGGCATTCACAGTGGCTAAG, and miR-27a R: GTGCAGGGTCCGAGGT; miR-195 RT primer: GTCGTATCCAGTGCAGGGTCCGAGGTATTCGCACTGGATACGACGCCAAT, miR-195 F: TAGCAGCACAGAAAT, and miR-195 R: GTGCAGGGTCCGAGGT; and miR-199a RT primer: GTCGTATCCAGTGCAGGGTCCGAGGTATTCGCACTGGATACGACGAACAG, miR-199a F: CTGAGTCCCAGTGTTCAGACT, and miR-199 R: GTGCAGGGTCCGAGGT.

### 2.4 Luciferase reporter assay

The 3’ UTR of YAP1 was amplified by PCR from human cDNA. The primer sequences and product sizes were as follows: Xho1 YAP1-3’ UTR F: TCGCTCGAGAGCCCTCAGGCAGACTGAATTC and Not1 YAP1-3’ UTR R: CCAGCGGCCGCGCACTAGGAGGAAAAGAAAATA, size: 566 bp. Overlap PCR was used to establish the YAP1 3’ UTR mutant plasmid by deleting the sequence, UGCUGCUG, that might pair with the miR-195 seed sequence. The PCR products were digested with XhoI and Not1, and cloned into the 3’ UTR of Renilla luciferase in the psiCheck2 plasmid. 293T cells were co-transfected with the YAP1 3’ UTR (wild-type or mutant) luciferase reporter plasmid and miRNA-expressing plasmids. After 24 hours, cells were lysed, and luciferase activity was measured using the Dual-Luciferase Reporter Assay System (Promega). Firefly luciferase activity was used for normalization.

### 2.5 Western blot analysis

Total protein was extracted and separated by SDS-PAGE, then transferred to PVDF membranes (Millipore). Membranes were blocked with 5% non-fat milk and incubated overnight with primary antibodies against YAP1 (CST, #14074) or GAPDH (CST, #5174). HRP-conjugated secondary antibodies were used, and signals were detected using ECL reagents (Amersham).

### 2.6 Lentivirus production and infection

Recombinant lentiviruses expressing miR-195 or control vectors were produced by co-transfecting 293T cells with pCDH plasmids, pMD.G, and psPAX2. Viral supernatants were collected at 24 and 48 hours post-transfection and used to infect target cells.

### 2.7 Cell viability and colony formation assays

Cell viability was assessed using the CCK-8 assay (Dojindo) at 0, 24, and 48 hours post-transfection. For colony formation, 5000 cells were seeded in 10-cm dishes and cultured for 10 days. Colonies were fixed with methanol, stained with crystal violet, and counted using ImageJ software.

### 2.8 Tumor xenografts

All animal procedures were approved by the Institutional Animal Care and Use Committee of the Second Military Medical University (Approval No. SMMU310528). HCT116 cells (5 × 10^6) stably expressing miR-195 or control vectors were injected subcutaneously into the flanks of nude mice (n=6 per group). Tumor dimensions were measured every 7 days using calipers, and volumes were calculated.

### 2.9 Statistical analysis

Data are presented as mean ± SD. Differences between two groups were analyzed using the two-tailed unpaired Student’s t-test. One-way ANOVA was used for comparisons among multiple groups. *P* < 0.05 was considered statistically significant.

## 3 Results

### 3.1 MiR-195 exhibits the strongest suppressive effect on YAP1 3’ UTR activity

**Figure 1:**
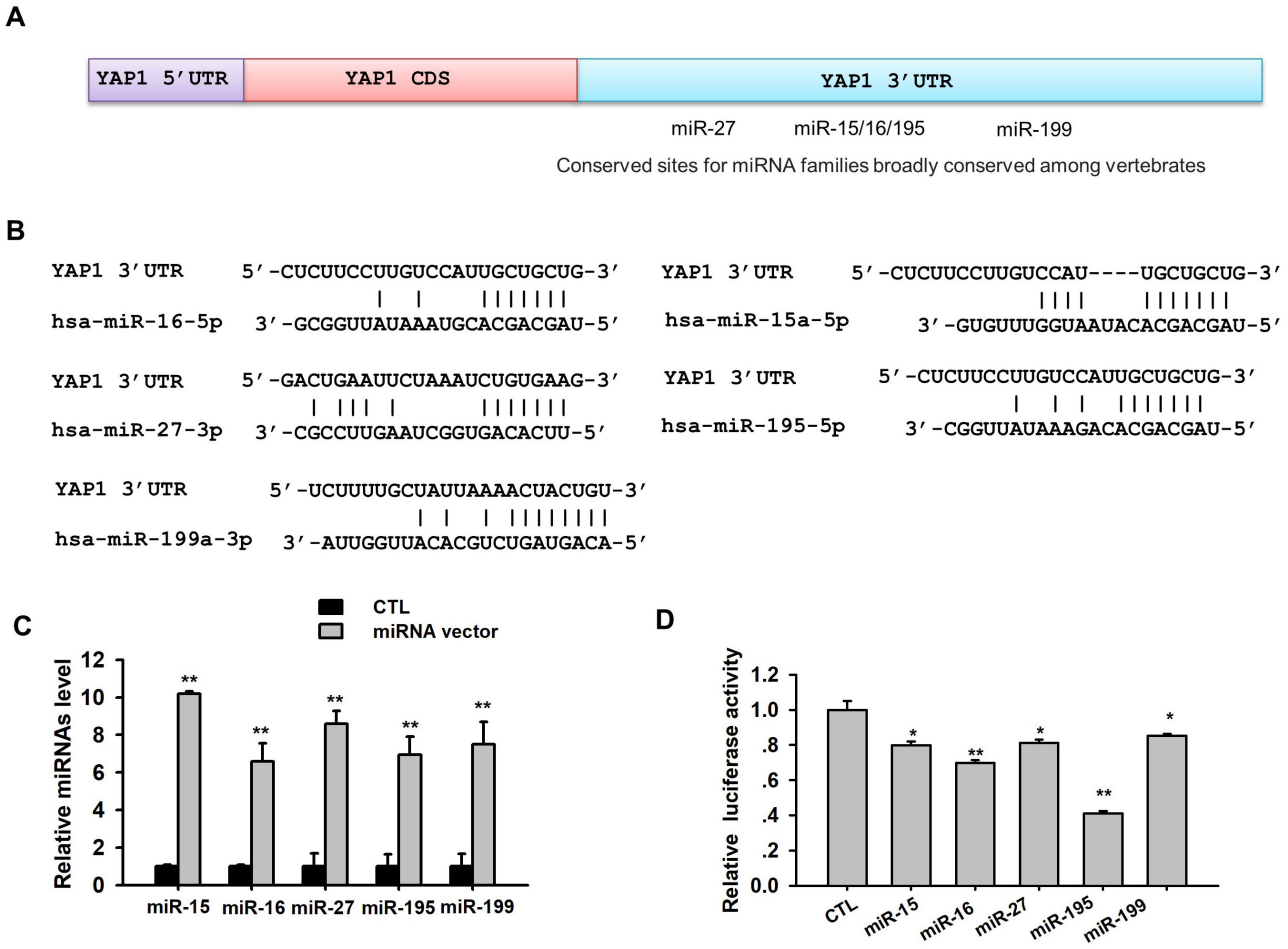
Systematic evaluation of miRNA-mediated repression of YAP1 via luciferase reporter assays. **(A)** MiR-15, miR-16, miR-27, miR-195, and miR-199 were predicted to directly target the 3’ UTR sequence of YAP1. **(B)** Sequence alignment analysis demonstrating the complementarity between the seed regions of the indicated miRNAs and the YAP1 3’ UTR. **(C)** qRT-PCR validation confirming approximately 10-fold overexpression of the respective miRNAs in transfected cells compared to the control. **(D)** Overexpression of all tested miRNAs significantly suppressed *Renilla* luciferase activity. MiR-15, miR-27, and miR-199 exhibited mild suppression (~20%), miR-16 showed moderate suppression (~30%), and miR-195 demonstrated the strongest suppression (~60%). * p < 0.05, ** p < 0.01.

To identify miRNAs capable of regulating YAP1 expression, we performed a luciferase reporter screen. Bioinformatic analysis using TargetScan and miRanda predicted five miRNAs (miR-15, miR-16, miR-27, miR-195, and miR-199) with potential binding sites within the YAP1 3’ untranslated region (3’ UTR) (**Figures 1A, B**). To validate these predictions, we cloned the wild-type YAP1 3’ UTR downstream of the Renilla luciferase gene in the psiCheck2 vector. 293T cells were co-transfected with this reporter construct and plasmids expressing individual miRNAs (**Figure 1C**). As shown in **Figure 1D**, all five miRNAs significantly suppressed luciferase activity compared to the control group, confirming their ability to bind the YAP1 3’ UTR. However, miR-195 exhibited the most robust inhibitory effect, reducing luciferase activity by approximately 60%. In contrast, miR-15, miR-27, and miR-199 induced a moderate reduction of roughly 20%, while miR-16 resulted in a 30% suppression. Based on this superior efficacy, we selected miR-195 for further mechanistic and functional characterization.

### 3.2 MiR-195 directly binds to the YAP1 3’ UTR to suppress protein expression

**Figure 2:**
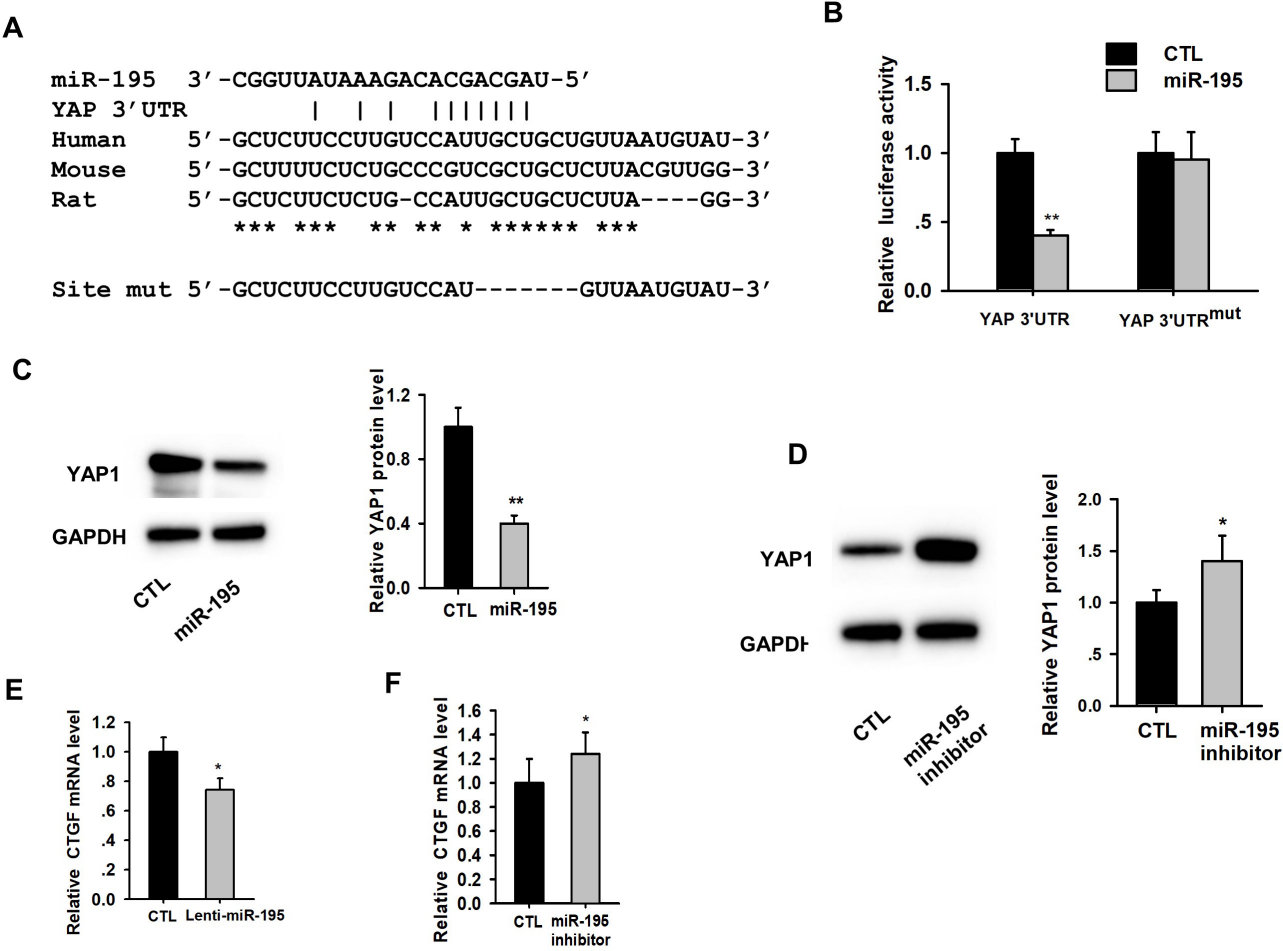
MiR-195 directly targets the YAP1 3’ UTR. **(A)** Schematic representation of the mutagenesis strategy. A mutant *Renilla* luciferase reporter plasmid was generated by deleting the sequence complementary to the miR-195 seed region within the YAP1 3’ UTR. **(B)** Luciferase assay results showing that the deletion of the seed sequence abolished the suppressive effect of miR-195. **(C, D)** Western blot analysis confirmed that miR-195 overexpression reduced endogenous YAP1 protein levels, while miR-195 knockdown increased YAP1 expression. **(E, F)** qRT-PCR analysis revealed that miR-195 overexpression significantly down-regulated CTGF (a canonical transcriptional target of YAP1) **(E)**, while miR-195 knockdown markedly up-regulated CTGF **(F)**. * p < 0.05, ** p < 0.01. ***, ****** indicates nucleotides that are conserved across human, mouse, and rat species.

To confirm the direct and specific interaction between miR-195 and the YAP1 3’ UTR, we generated a mutant reporter construct in which the seed sequence (nucleotides 2-8) complementary to miR-195 was disrupted by site-directed mutagenesis (**Figure 2A**). Dual-luciferase assays revealed that while miR-195 overexpression significantly suppressed the activity of the wild-type YAP1 3’ UTR reporter, this suppressive effect was completely abrogated in the mutant construct (**Figure 2B**). This confirms that the observed repression is dependent on the specific seed sequence match.

We further validated this regulation at the protein level. Western blot analysis demonstrated that transient transfection of miR-195 overexpression plasmid in 293T cells led to a marked reduction in endogenous YAP1 protein levels (**Figures 2C, D**). Conversely, transfection of a miR-195 inhibitor (50 nM) significantly increased YAP1 protein expression compared to the negative control inhibitor group. Furthermore, consistent with YAP1 being a transcriptional co-activator, we observed a significant downregulation of *CTGF* (Connective Tissue Growth Factor), a well-characterized downstream target gene of YAP1, at the mRNA level upon miR-195 overexpression and knockdown (**Figures 2E, F**). These results demonstrate that miR-195 directly targets the YAP1 3’ UTR to suppress its translation and subsequent transcriptional activity.

### 3.3 MiR-195 suppressed tumor cell proliferation and clonogenic survival

**Figure 3:**
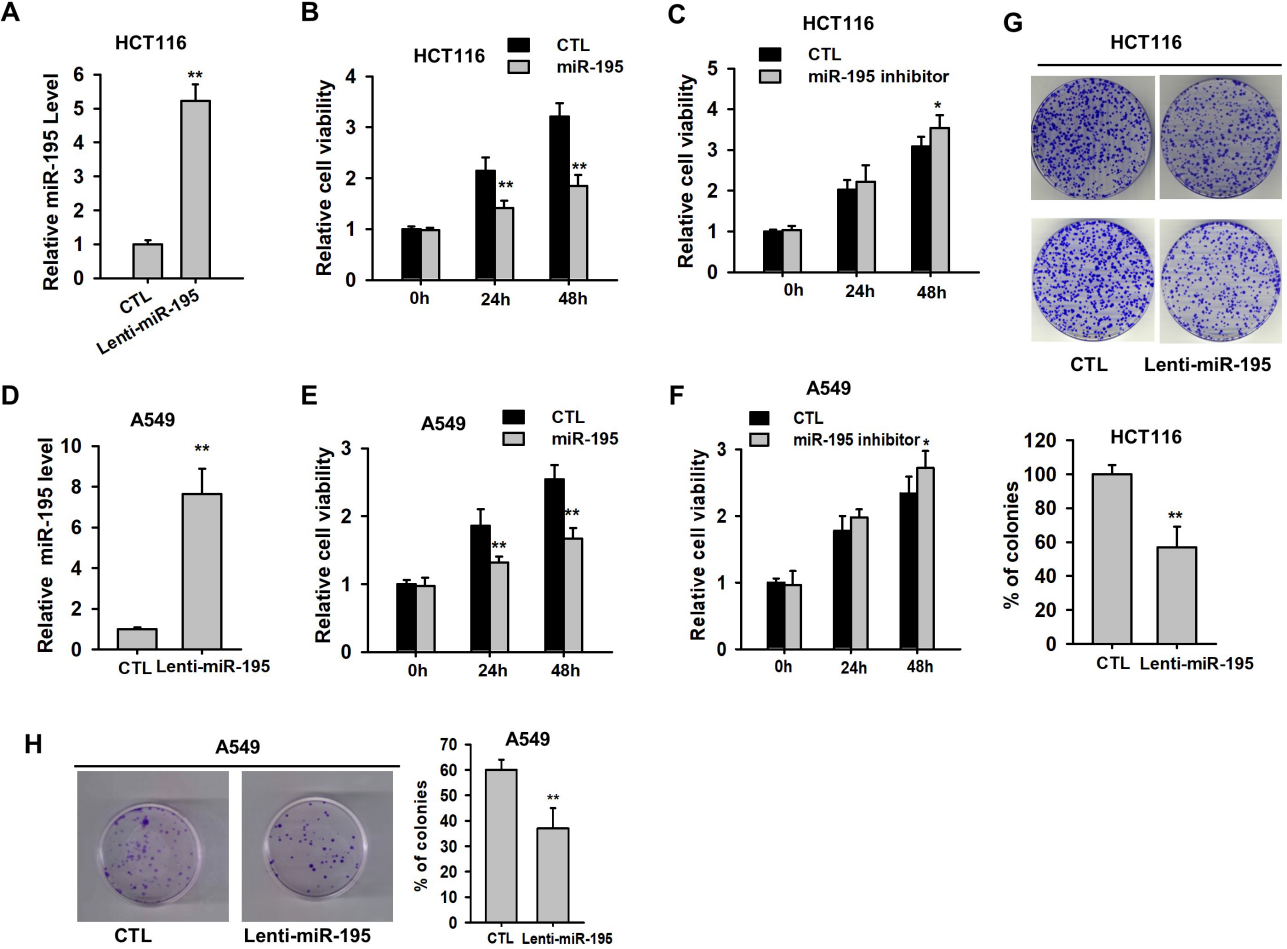
MiR-195 inhibits cell viability, proliferation, and colony formation. **(A, D)** qRT-PCR confirmed efficient overexpression and knockdown of miR-195 in HCT116 and A549 cell lines. **(B, C, E, F)** CCK-8 assays showed that miR-195 overexpression significantly reduced cell viability and proliferation, whereas miR-195 knockdown enhanced these processes. **(G, H)** Representative images and quantification showing that miR-195 overexpression decreased colony formation by approximately 40%. * p < 0.05, ** p < 0.01.

Given the established role of YAP1 in promoting cell proliferation (32), we next investigated the functional impact of miR-195 on tumor cell growth. We utilized two distinct human cancer cell lines: HCT116 (colorectal carcinoma) and A549 (lung adenocarcinoma). Cell Counting Kit-8 (CCK-8) assays showed that overexpression of miR-195 significantly impaired cell viability in both cell lines (**Figures 3A, D**). In HCT116 cells, viability was reduced by approximately 20% at 24 hours and 40% at 48 hours post-transfection compared to the control group (**Figures 3B, E**). Similar results were observed in A549 cells (**Figures 3C, F**). Conversely, knockdown of endogenous miR-195 using specific inhibitors promoted cell proliferation in both lines. To assess long-term proliferative capacity, we performed colony formation assays. HCT116 cells were transfected with lenti-miR-195 and seeded at low density. After 10 days, the number of visible colonies was quantified. As shown in **Figure 3G**, miR-195 overexpression resulted in a ~40% reduction in the number of colonies formed compared to the control. The same results were also identified in A549 colony formation assays (**Figure 3H**). These data indicate that miR-195 acts as a potent suppressor of tumor cell proliferation.

### 3.4 Rescue of proliferative defects by YAP1 overexpression confirms target specificity

**Figure 4:**
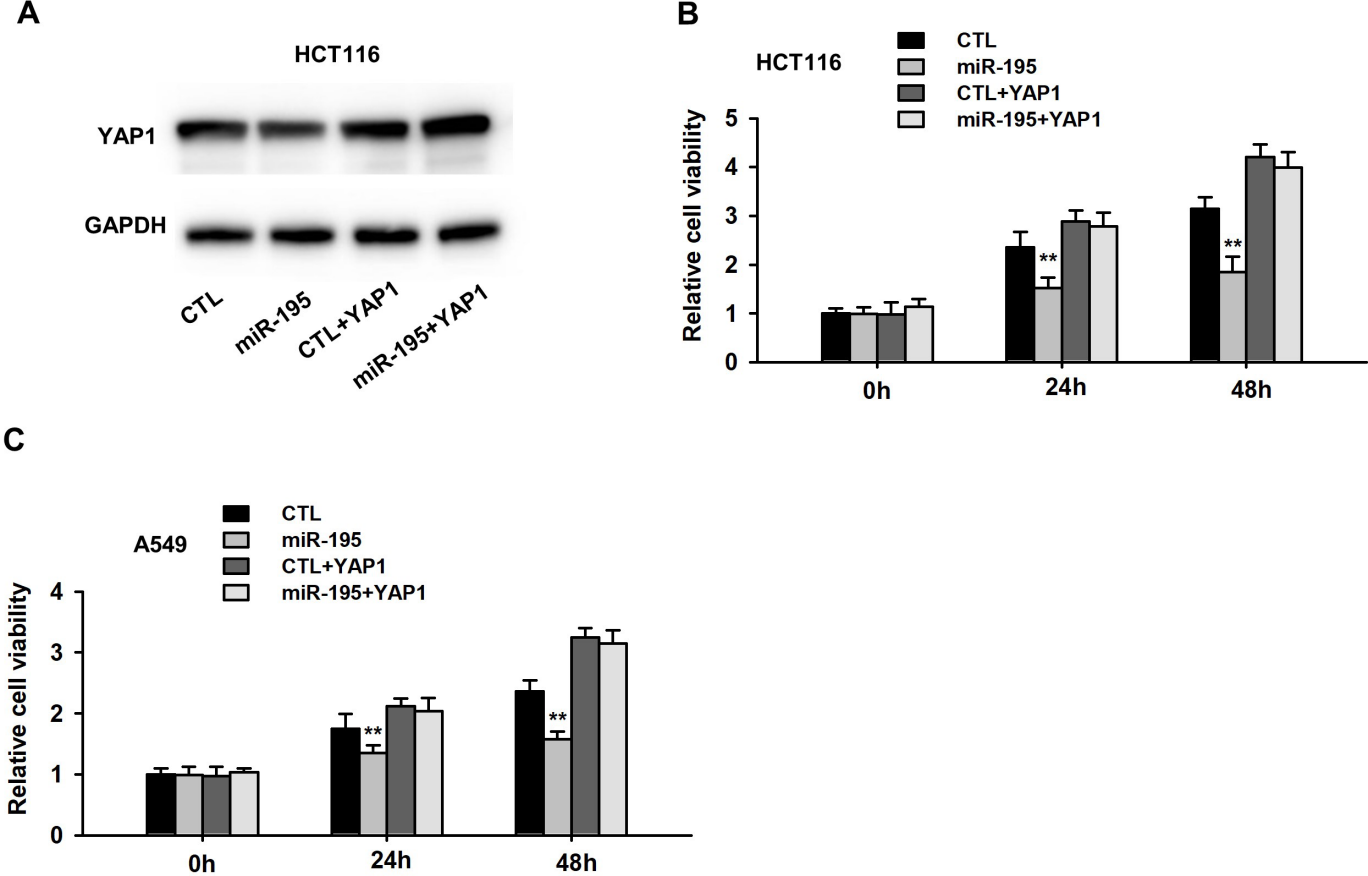
MiR-195 exerts its effects on cell proliferation through YAP1 suppression. **(A)** Western blot showing that miR-195 suppressed endogenous YAP1 expression, while lenti-YAP1 restored YAP1 protein levels. **(B, C)** Functional rescue assays demonstrated that while miR-195 overexpression inhibited cell viability and YAP1 overexpression promoted it, the co-overexpression of YAP1 and miR-195 resulted in cell viability similar to the YAP1-overexpression-alone group. This indicates that YAP1 overexpression counteracts the inhibitory effects of miR-195. ** P < 0.01.

To rigorously demonstrate that the anti-proliferative effects of miR-195 are mediated specifically through YAP1 suppression, we performed rescue experiments. We constructed a YAP1 expression vector lacking the 3’ UTR (pCMV-YAP1), rendering it insensitive to miRNA regulation. HCT116 and A549 cells were then co-transfected with either lenti-miR-195 or a negative control (**Figure 4A**), along with either the pCMV-YAP1 vector or an empty control vector. As expected, miR-195 overexpression alone significantly suppressed cell viability in both cell lines. However, when YAP1 was exogenously expressed alongside miR-195, the proliferative capacity of the cells was restored to levels comparable to the control group (**Figures 4B, C**). This indicates that the inhibitory effect of miR-195 on cell proliferation is primarily dependent on its ability to target and downregulate YAP1, rather than off-target effects.

### 3.5 MiR-195 suppresses tumor growth *in vivo* and correlates with clinical prognosis

**Figure 5:**
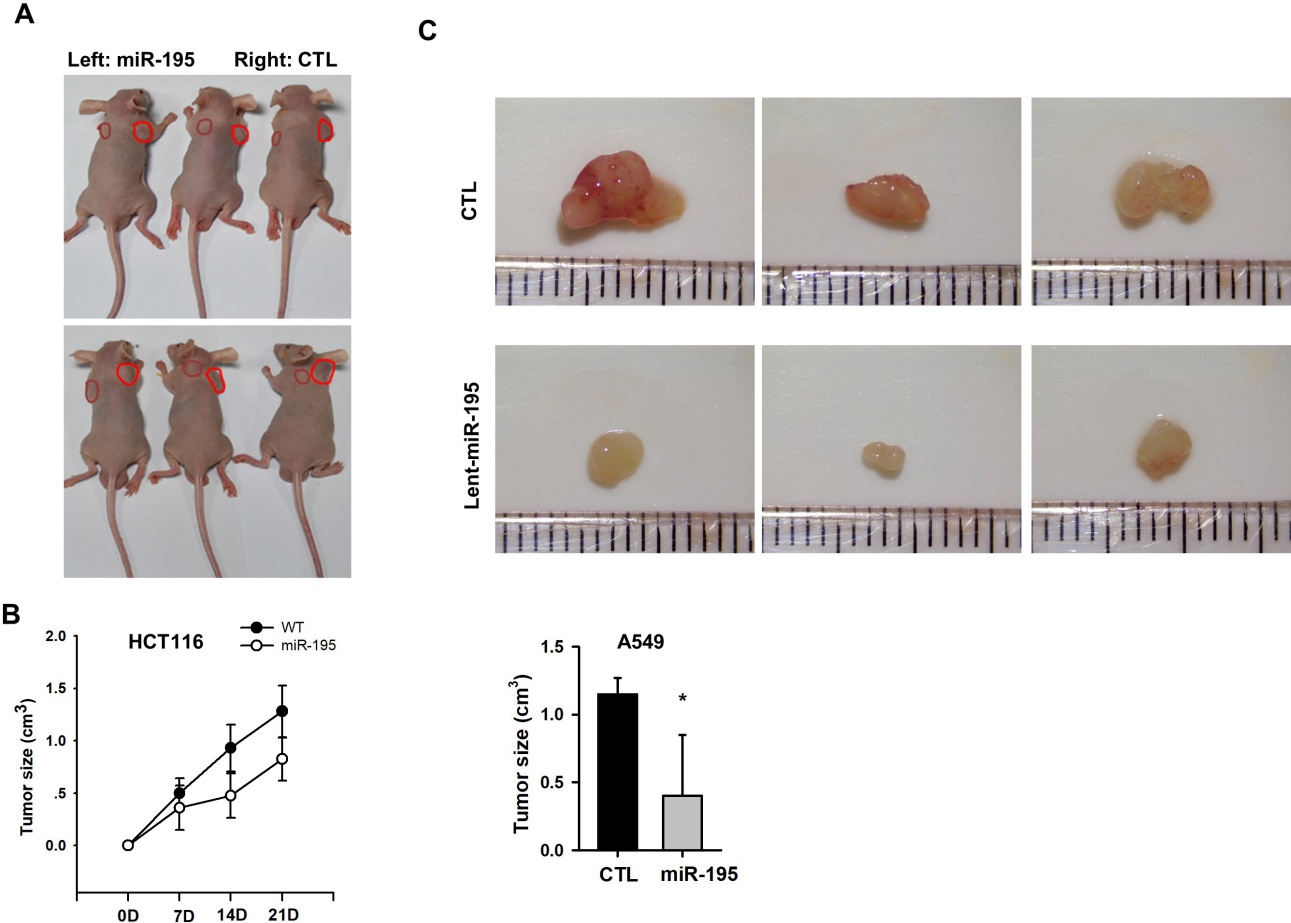
Overexpression of miR-195 suppresses tumor progression *in vivo*. **(A)** HCT116 cells (control on the right flank; miR-195-overexpressing on the left flank) were injected into immune-deficient mice. Representative images of tumors excised 21 days post-injection showing smaller tumor size in the miR-195 group. **(B)** Tumor growth curves indicated that miR-195-overexpressing tumors were significantly smaller than control tumors at both 7 and 14 days. (Black dots represent miR-195-overexpressing cells; white dots represent wild-type cells). **(C)** A549 cells were injected into immune-deficient mice. MiR-195 overexpression suppresses tumor progression.

To evaluate the therapeutic potential of miR-195 *in vivo*, we established a xenograft tumor model in nude mice. HCT116 cells stably overexpressing miR-195 (via lentiviral transduction) or control vectors were injected subcutaneously into the flanks of the mice. Tumor growth was monitored over a period of 21 days. As shown in **Figures 5A, B**, tumors derived from miR-195-overexpressing cells grew significantly slower than control tumors. At the experimental endpoint, the average tumor volume in the miR-195 group were significantly lower than those in the control group, confirming the tumor-suppressive role of miR-195 *in vivo*. The same results were observed in A549 cells (**Figure 5C**).

**Figure 6:**
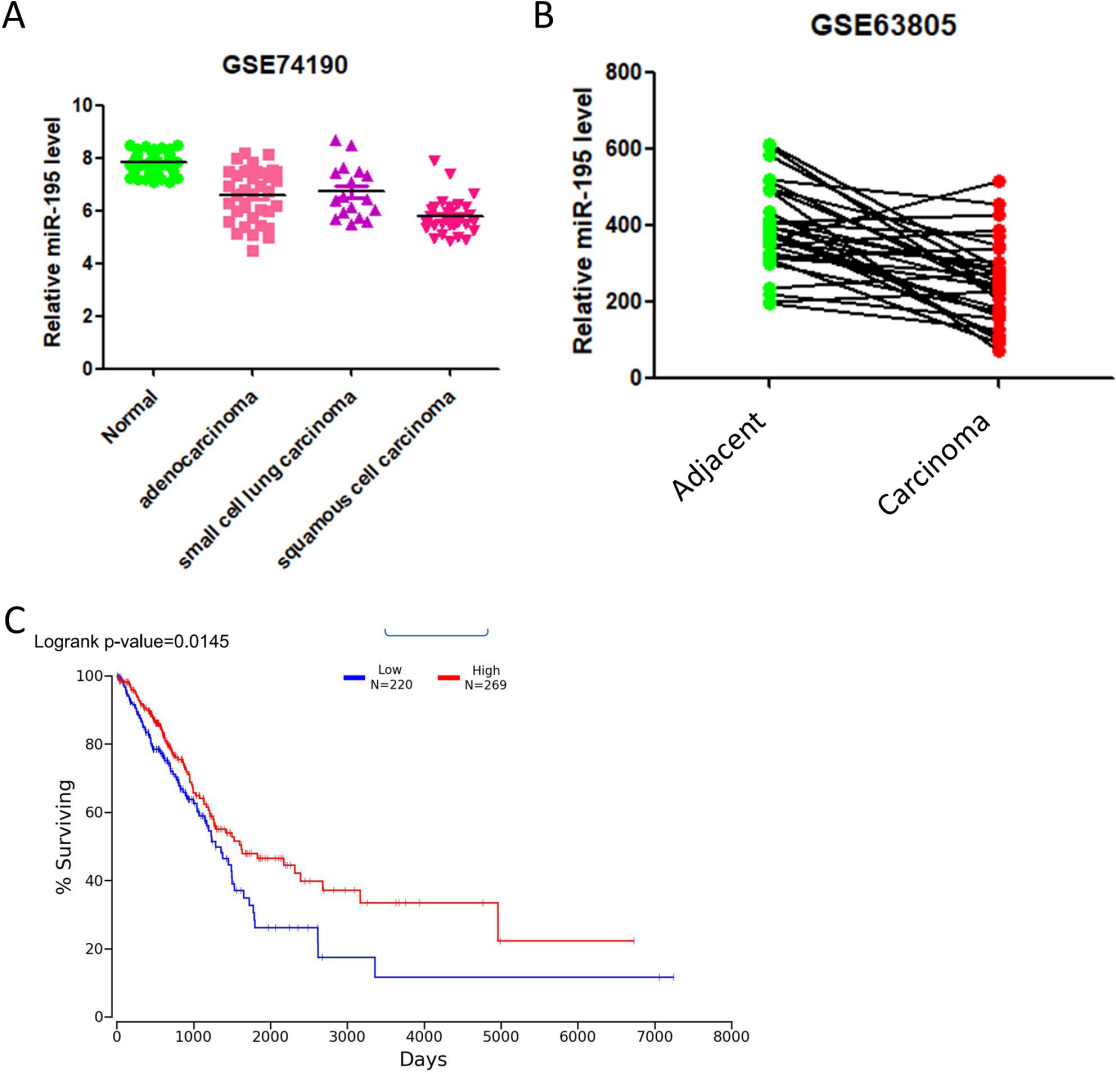
Clinical correlation of miR-195 expression in lung cancer. **(A, B)** Statistical analysis confirmed lower miR-195 levels in lung carcinoma using two independent Gene Expression Omnibus (GEO) datasets, GSE74190 and GSE63805. MiR-195 expression was significantly downregulated in lung tumor tissues compared to adjacent normal tissues. **(C)** Kaplan-Meier survival analysis revealed that lower miR-195 expression was associated with poor survival outcomes in lung cancer patients.

Finally, to evaluate the clinical significance of our findings, we investigated the expression levels of miR-195 in various human tumor tissues. To further validate these observations, we performed a comprehensive analysis using two independent Gene Expression Omnibus (GEO) datasets, GSE74190 and GSE63805. Quantitative analysis revealed that miR-195 expression was significantly downregulated in tumor tissues compared to adjacent non-tumor tissues, with the most pronounced reduction observed in lung carcinoma samples (**Figures 6A, B**). Kaplan-Meier survival analysis further demonstrated that patients with lower miR-195 expression levels exhibited significantly poorer overall survival rates (**Figure 6C**). These clinical data corroborate our experimental findings, suggesting that loss of miR-195 expression contributes to tumor progression and poor prognosis in human cancers.

## 4 Discussion

It has been reported that multiple miRNAs had the ability to suppress the expression of a common target gene (22). This study provides compelling evidence that microRNAs (miRNAs) are critical regulators of YAP1 expression and, consequently, key modulators of tumor progression. We evaluated five candidate miRNAs, miR-15, miR-16, miR-27, miR-195 and miR-199. All of these five miRNAs demonstrated the ability to suppress luciferase activity when fused with the YAP1 3’ UTR, confirming their capacity to interact with this region. The observation that multiple miRNAs can converge on a single target gene like YAP1 is consistent with the concept of combinatorial gene regulation, which allows for robust and nuanced control of gene expression. However, the degree of suppression varied markedly. This variation may be attributed to differences in the specific seed sequence matching, the local RNA secondary structure of the YAP1 3’ UTR, or the relative abundance of RNA-binding proteins that might sterically hinder or facilitate miRNA access (33, 34). While our study focused on individual miRNA effects, it would be highly informative to explore the synergistic potential of combining these miRNAs. For instance, a cocktail of miR-195, miR-16, and others might exert a more potent suppressive effect on YAP1 than any single miRNA alone, potentially offering a more effective strategy for tumor suppression with lower dosing requirements and reduced off-target effects.

Among the candidates, miR-195 emerged as the most potent suppressor of the YAP1 3’ UTR-driven reporter. This functional efficacy aligns with its observed role in inhibiting tumor progression, suggesting a direct link between YAP1 downregulation and reduced malignancy. Similarly, miR-16, which has been previously documented as a tumor suppressor (35), also showed significant repression of the YAP1 reporter, reinforcing its role in cancer biology. However, our findings regarding miR-199 present a fascinating paradox. While it effectively suppressed the YAP1 3’ UTR reporter, its overexpression did not inhibit tumor progression; in fact, it appeared to facilitate it. Further analysis revealed that miR-199 up-regulates a network of cancer-related genes (36). This suggests that although YAP1 is a direct target, it is not the dominant functional target in the context of tumorigenesis. The oncogenic phenotype driven by the upregulation of other genes appears to outweigh the benefits of YAP1 suppression. This underscores a critical principle in miRNA therapeutics: the functional outcome of miRNA modulation depends on the net effect across its entire targetome, not just on individual key genes.

The complexity of miRNA regulation is further exemplified by the diverse set of validated targets for miR-195, including *MYB* proto-oncogene, transcription factor (*MYB*) (37), ADP ribosylation factor like GTPase 2 (*ARL2*) (38), fatty acid synthase (*FASN*) (39), BCL2, apoptosis regulator (*BCL2*) (40), cyclin E1 (*CCNE1*) (41), and cyclin D1 (*CCND1*) (41). Many of these genes are themselves involved in cell proliferation, survival, and metabolism. The dysregulation of miR-195-5p appears to be a hallmark of aggressive disease across these malignancies, functioning as a critical node in a complex regulatory network that governs cell proliferation, metastasis, and therapeutic resistance (42, 43). In colorectal cancer, it acts as a master regulator of epithelial integrity and cytoskeletal dynamics by directly targeting *KRT80*, *KRT23*, and components of the adherens junctions, thereby suppressing invasion and maintaining tissue architecture (44). In lung cancer, its tumor-suppressive function is mediated through the inhibition of redox metabolism and the modulation of oncogenic signaling pathways (45). Furthermore, the clinical relevance of miR-195-5p is underscored by its involvement in chemoresistance. Therefore, the anti-tumor effects of miR-195 are likely the result of a coordinated attack on multiple oncogenic pathways simultaneously, with YAP1 being one crucial component of this network (46). Our study reinforces the pivotal role of miR-195-5p as a broad-spectrum tumor suppressor, demonstrating significant implications for both colorectal cancer and lung cancer pathogenesis. The consistent downregulation of miR-195-5p in both tumor types, often associated with advanced staging and poor prognosis, highlights its potential as a promising candidate for future nucleic acid-based therapeutics.

Beyond cancer, YAP1 is a master regulator of organ size, tissue regeneration, and stem cell maintenance. Given the potency with which these miRNAs down-regulate YAP1, it is worth investigating their roles in developmental biology and regenerative medicine. For example, the heart failure phenotype observed in miR-195 transgenic mice could potentially be linked to excessive suppression of YAP1, which is known to be vital for cardiomyocyte proliferation and heart growth (47). Exploring this connection could open new avenues for treating degenerative diseases.

In conclusion, our work identifies a network of miRNAs capable of regulating YAP1, with miR-195 standing out as a particularly effective tumor suppressor. The therapeutic potential of these miRNAs, either as single agents or in combination, warrants systematic evaluation. Future research should focus not only on maximizing target gene suppression but also on understanding the holistic impact of miRNA modulation on the entire gene regulatory network to ensure safe and effective clinical translation.

## Data Availability

The original contributions presented in the study are included in the article/supplementary material. Further inquiries can be directed to the corresponding author.

## References

[1] Piccolo S Dupont S Cordenonsi M . The biology of YAP/TAZ: hippo signaling and beyond. Physiol Rev. (2014) 94:1287–312. doi: 10.1152/physrev.00005.2014 25287865

[2] Oka T Mazack V Sudol M . Mst2 and Lats kinases regulate apoptotic function of Yes kinase-associated protein (YAP). J Biol Chem. (2008) 283:27534–46. doi: 10.1074/jbc.m804380200 18640976

[3] Kanai F Marignani PA Sarbassova D Yagi R Hall RA Donowitz M . TAZ: a novel transcriptional co-activator regulated by interactions with 14-3-3 and PDZ domain proteins. EMBO J. (2000) 19:6778–91. doi: 10.1093/emboj/19.24.6778 11118213 PMC305881

[4] Zhao B Li L Tumaneng K Wang CY Guan KL . A coordinated phosphorylation by Lats and CK1 regulates YAP stability through SCF(beta-TRCP). Genes Dev. (2010) 24:72–85. doi: 10.1101/gad.1843810 20048001 PMC2802193

[5] Hong W . Angiomotin'g YAP into the nucleus for cell proliferation and cancer development. Sci Signaling. (2013) 6:pe27. doi: 10.1126/scisignal.2004573 24003252

[6] Zhao Y Khanal P Savage P She YM Cyr TD Yang X . YAP-induced resistance of cancer cells to antitubulin drugs is modulated by a Hippo-independent pathway. Cancer Res. (2014) 74:4493–503. doi: 10.1158/0008-5472.can-13-2712 24812269

[7] Lamar JM Stern P Liu H Schindler JW Jiang ZG Hynes RO . The Hippo pathway target, YAP, promotes metastasis through its TEAD-interaction domain. PNAS. (2012) 109:E2441–2450. doi: 10.1073/pnas.1212021109 22891335 PMC3443162

[8] Chan SW Lim CJ Chen L Chong YF Huang C Song H . The Hippo pathway in biological control and cancer development. J Cell Physiol. (2011) 226:928–39. doi: 10.1002/jcp.22435 20945341

[9] Moroishi T Hansen CG Guan KL . The emerging roles of YAP and TAZ in cancer. Nat Rev Cancer. (2015) 15:73–9. doi: 10.1038/nrc3876 25592648 PMC4562315

[10] Dong J Feldmann G Huang J Wu S Zhang N Comerford SA . Elucidation of a universal size-control mechanism in Drosophila and mammals. Cell. (2007) 130:1120–33. doi: 10.1016/j.cell.2007.07.019 17889654 PMC2666353

[11] Bui DA Lee W White AE Harper JW Schackmann RC Overholtzer M . Cytokinesis involves a nontranscriptional function of the Hippo pathway effector YAP. Sci Signaling. (2016) 9:ra23. doi: 10.1126/scisignal.aaa9227 26933062 PMC5455055

[12] Yuan M Tomlinson V Lara R Holliday D Chelala C Harada T . Yes-associated protein (YAP) functions as a tumor suppressor in breast. Cell Death Differ. (2008) 15:1752–9. doi: 10.1038/cdd.2008.108 18617895

[13] Yagi R Chen LF Shigesada K Murakami Y Ito Y . A WW domain-containing yes-associated protein (YAP) is a novel transcriptional co-activator. EMBO J. (1999) 18:2551–62. doi: 10.1093/emboj/18.9.2551 10228168 PMC1171336

[14] Stein C Bardet AF Roma G Bergling S Clay I Ruchti A . YAP1 exerts its transcriptional control via TEAD-mediated activation of enhancers. PloS Genet. (2015) 11:e1005465. doi: 10.1371/journal.pgen.1005465 26295846 PMC4546604

[15] Liu-Chittenden Y Huang B Shim JS Chen Q Lee SJ Anders RA . Genetic and pharmacological disruption of the TEAD-YAP complex suppresses the oncogenic activity of YAP. Genes Dev. (2012) 26:1300–5. doi: 10.1101/gad.192856.112 22677547 PMC3387657

[16] Zanconato F Forcato M Battilana G Azzolin L Quaranta E Bodega B . Genome-wide association between YAP/TAZ/TEAD and AP-1 at enhancers drives oncogenic growth. Nat Cell Biol. (2015) 17:1218–27. doi: 10.1038/ncb3216 26258633 PMC6186417

[17] Pefani DE Pankova D Abraham AG Grawenda AM Vlahov N Scrace S . TGF-beta targets the Hippo pathway scaffold RASSF1A to facilitate YAP/SMAD2 nuclear translocation. Mol Cell. (2016) 63:156–66. doi: 10.1016/j.molcel.2016.05.012 27292796

[18] Okazaki T Kageji T Kuwayama K Kitazato KT Mure H Hara K . Up-regulation of endogenous PML induced by a combination of interferon-beta and temozolomide enhances p73/YAP-mediated apoptosis in glioblastoma. Cancer Lett. (2012) 323:199–207. doi: 10.1016/j.canlet.2012.04.013 22542810

[19] Yang WJ Yang DD Na S Sandusky GE Zhang Q Zhao G . Dicer is required for embryonic angiogenesis during mouse development. J Biol Chem. (2005) 280:9330–5. doi: 10.1074/jbc.m413394200 15613470

[20] Meister G Landthaler M Patkaniowska A Dorsett Y Teng G Tuschl T . Human Argonaute2 mediates RNA cleavage targeted by miRNAs and siRNAs. Mol Cell. (2004) 15:185–97. doi: 10.1016/j.molcel.2004.07.007 15260970

[21] Friedman RC Farh KK Burge CB Bartel DP . Most mammalian mRNAs are conserved targets of microRNAs. Genome Res. (2009) 19:92–105. doi: 10.1101/gr.082701.108 18955434 PMC2612969

[22] Li M Marin-Muller C Bharadwaj U Chow KH Yao Q Chen C . MicroRNAs: control and loss of control in human physiology and disease. World J Surg. (2009) 33:667–84. doi: 10.1007/s00268-008-9836-x 19030926 PMC2933043

[23] Ha TY . MicroRNAs in human diseases: from cancer to cardiovascular disease. Immune Network. (2011) 11:135–54. doi: 10.4110/in.2011.11.3.135 21860607 PMC3153666

[24] Song XW Li Q Lin L Wang XC Li DF Wang GK . MicroRNAs are dynamically regulated in hypertrophic hearts, and miR-199a is essential for the maintenance of cell size in cardiomyocytes. J Cell Physiol. (2010) 225:437–43. doi: 10.1002/jcp.22217 20458739

[25] Cai ZG Zhang SM Zhang H Zhou YY Wu HB Xu XP . Aberrant expression of microRNAs involved in epithelial-mesenchymal transition of HT-29 cell line. Cell Biol Int. (2013) 37:669–74. doi: 10.1002/cbin.10087 23483606

[26] Hua K Jin J Zhao J Song J Song H Li D . miR-135b, upregulated in breast cancer, promotes cell growth and disrupts the cell cycle by regulating LATS2. Int J Oncol. (2016) 48:1997–2006. doi: 10.3892/ijo.2016.3405 26934863

[27] Higashi T Hayashi H Ishimoto T Takeyama H Kaida T Arima K . miR-9-3p plays a tumour-suppressor role by targeting TAZ (WWTR1) in hepatocellular carcinoma cells. Br J Cancer. (2015) 113:252–8. doi: 10.1038/bjc.2015.170 26125451 PMC4506379

[28] Nandy SB Arumugam A Subramani R Pedroza D Hernandez K Saltzstein E . MicroRNA-125a influences breast cancer stem cells by targeting leukemia inhibitory factor receptor which regulates the Hippo signaling pathway. Oncotarget. (2015) 6:17366–78. doi: 10.18632/oncotarget.3953 25962054 PMC4627314

[29] Zeng G Xun W Wei K Yang Y Shen H . MicroRNA-27a-3p regulates epithelial to mesenchymal transition via targeting YAP1 in oral squamous cell carcinoma cells. Oncol Rep. (2016) 36:1475–82. doi: 10.3892/or.2016.4916 27432214

[30] Kang W Tong JH Lung RW Dong Y Zhao J Liang Q . Targeting of YAP1 by microRNA-15a and microRNA-16-1 exerts tumor suppressor function in gastric adenocarcinoma. Mol Cancer. (2015) 14:52. doi: 10.1186/s12943-015-0323-3 25743273 PMC4342823

[31] Ren K Li T Zhang W Ren J Li Z Wu G . miR-199a-3p inhibits cell proliferation and induces apoptosis by targeting YAP1, suppressing Jagged1-Notch signaling in human hepatocellular carcinoma. J BioMed Sci. (2016) 23:79. doi: 10.1186/s12929-016-0295-7 27832779 PMC5103406

[32] Zhou S Chen HZ Wan YZ Zhang QJ Wei YS Huang S . Repression of P66Shc expression by SIRT1 contributes to the prevention of hyperglycemia-induced endothelial dysfunction. Circ Res. (2011) 109:639–48. doi: 10.1161/circresaha.111.243592 21778425

[33] Kim S Kim S Chang HR Kim D Park J Son N . The regulatory impact of RNA-binding proteins on microRNA targeting. Nat Commun. (2021) 12:5057. doi: 10.1038/s41467-021-25078-5 34417449 PMC8379221

[34] Yang M Woolfenden HC Zhang Y Fang X Liu Q Vigh ML . Intact RNA structurome reveals mRNA structure-mediated regulation of miRNA cleavage *in vivo*. Nucleic Acids Res. (2020) 48:8767–81. doi: 10.1093/nar/gkaa577 32652041 PMC7470952

[35] Wang W Chen J Dai J Zhang B Wang F Sun Y . MicroRNA-16-1 inhibits tumor cell proliferation and induces apoptosis in A549 non-small cell lung carcinoma cells. Oncol Res. (2016) 24:345–51. doi: 10.3727/096504016x14685034103194 27712591 PMC7838694

[36] Wei W Wang L Xu L Liang J Teng L . MiR-199 reverses the resistance to gemcitabine in pancreatic cancer by suppressing stemness through regulating the epithelial-mesenchymal transition. ACS Omega. (2021) 6:31435–46. doi: 10.1021/acsomega.1c02945 34869970 PMC8637594

[37] Yongchun Z Linwei T Xicai W Lianhua Y Guangqiang Z Ming Y . MicroRNA-195 inhibits non-small cell lung cancer cell proliferation, migration and invasion by targeting MYB. Cancer Lett. (2014) 347:65–74. doi: 10.1016/j.canlet.2014.01.019 24486218

[38] Zhou Y Jiang H Gu J Tang Y Shen N Jin Y . MicroRNA-195 targets ADP-ribosylation factor-like protein 2 to induce apoptosis in human embryonic stem cell-derived neural progenitor cells. Cell Death Dis. (2013) 4:e695. doi: 10.1038/cddis.2013.195 23807224 PMC3702293

[39] Singh R Yadav V Kumar S Saini N . MicroRNA-195 inhibits proliferation, invasion and metastasis in breast cancer cells by targeting FASN, HMGCR, ACACA and CYP27B1. Sci Rep. (2015) 5:17454. doi: 10.1038/srep17454 26632252 PMC4668367

[40] Chen YQ Wang XX Yao XM Zhang DL Yang XF Tian SF . MicroRNA-195 promotes apoptosis in mouse podocytes via enhanced caspase activity driven by BCL2 insufficiency. Am J Nephrol. (2011) 34:549–59. doi: 10.1159/000333809 22123611

[41] Hui W Yuntao L Lun L WenSheng L ChaoFeng L HaiYong H . MicroRNA-195 inhibits the proliferation of human glioma cells by directly targeting cyclin D1 and cyclin E1. PloS One. (2013) 8:e54932. doi: 10.1371/journal.pone.0054932 23383003 PMC3557299

[42] Alexandre D Baptista PV Cruz C . Harnessing microRNAs in lung cancer: the future of diagnosis and precision therapy. Biochim Biophys Acta Rev Cancer. (2026) 1881:189535. doi: 10.1016/j.bbcan.2026.189535 41579972

[43] Piccinno E Aloisio M Scalavino V Russo F Giannelli G Guido D . Exploring miRNA research in colorectal cancer: insights from a bibliometric analysis. Pharmaceutics. (2025) 17(8):1084. doi: 10.3390/pharmaceutics17081084 40871106 PMC12389379

[44] Piccinno E Scalavino V Labarile N Bianco G Armentano R Giannelli G . miR-195-5p suppresses KRT80 expression inducing cell cycle arrest in colon cancer. Cancers (Basel). (2025) 17(13):2183. doi: 10.3390/cancers17132183 40647481 PMC12248558

[45] Tomioka Y Seki N Mizuno K Suetsugu T Tsuruzono K Hagihara Y . MicroRNA signatures in lung adenocarcinoma metastases: exploring the oncogenic targets of tumor-suppressive miR-195-5p and miR-195-3p. Cancers (Basel). (2025) 17(14):2348. doi: 10.3390/cancers17142348 40723231 PMC12293978

[46] Liu SH Hsu KW Lai YL Lin YF Chen FH Peng PH . Systematic identification of clinically relevant miRNAs for potential miRNA-based therapy in lung adenocarcinoma. Mol Ther Nucleic Acids. (2021) 25:1–10. doi: 10.1016/j.omtn.2021.04.020 34141460 PMC8181588

[47] van Rooij E Sutherland LB Liu N Williams AH McAnally J Gerard RD . A signature pattern of stress-responsive microRNAs that can evoke cardiac hypertrophy and heart failure. PNAS. (2006) 103:18255–60. doi: 10.1073/pnas.0608791103 17108080 PMC1838739

